# It's Hard Work Being No One

**DOI:** 10.3389/fpsyg.2018.02632

**Published:** 2018-12-21

**Authors:** J. Scott Jordan

**Affiliations:** Department of Psychology, Director, Institute for Prospective Cognition, Illinois State University, Normal, IL, United States

**Keywords:** embodiment, Wild Systems Theory, self-sustaining systems, representation, naturalism, self-model


It's hard work Being No One
Trying so hard to erase
     the indelible trace
     the sun leaves on my face
 
It's hard work Being No One
Trying so hard to forget the HEs and SHEs
     and the THEYs and the WEs
     and the glorious YOUs and MEs
 
I got a ticket on the next train to OZ
Gotta see that crazy wizard because
     even tin man was an I, just had no heart
     scarecrow was an I, just not that smart
       and the lion, he's a coward, but at least he's an I
       me, I'm just a guy who has to deny he's an I
     oh I wish that I could depart
     this lonely land of Descartes
 
It's hard work Being No One
So why don't we
     just agree
     to find our Is and WEs
     in the midst of you and me
 
It's hard work Being No One
So why don't we just start a religion
     live in contradiction
     and sustain our addiction
 
to wanting to be
to wanting to be
wanting to be
just wanting to be
Someone


I wrote this song (It's Hard Work Being No One, Supplementary Audio 1) for Thomas Metzinger during the winter of 2006. We were part of a research team investigating *Embodied Communication in Humans and Machines*, organized by Ipke Wachsmuth and Guenther Knoblich. And while the song might seem a criticism of Thomas' *self-model theory of subjectivity* (SMTS—Metzinger, 2003), actually, it's a tribute to the most scientifically informed position on consciousness and the self, to date.

*It's hard* ***work*** *Being No One*. The song is also an Eric Idle-esque “wink-wink, nudge-nudge” at differences that exist between SMTS and Wild Systems Theory (WST—Jordan and Ghin, 2006; Jordan, 2013). WST follows the lead of Odum (1988) and conceptualizes living systems as multi-scale nestings of self-sustaining, energy-transformation systems. Such systems are self-sustaining because their *work* (i.e., energy expenditures) produces products (e.g., catalysts) that feedback into and, ultimately *sustain* the work. This principle has been discovered at many levels of scale, including chemical systems (*autocatalysis*—Kauffman, 1995), the single-cell (*autopoiesis*—Maturana and Varela, 1980), neural networks (*the cell assembly*—Hebb, 1949), behavior (*reinforcement theory*—Skinner, 1974), and ecologies in general (Odum, 1988).

*The indelible trace the sun leaves on* ***my*** *face*. Self-sustaining systems persist because their *work* generates and maintains permeable borders. In the case of a neuron, the border is a lipid bilayer. In the case of a conscious *self*, the border is a phenomenal self-model (PSM—Metzinger, 2003), that entails transparent, representational content of a, “…preattentive self-world border” (p. 307).

While SMTS and WST agree the generation and sustainment of a PSM border affords the existence of “mineness,” their differing approaches to “representation” lead to divergent accounts of why “mineness” entails the conscious sense of being *someone*.

*The lonely land of Descartes*. Theories of how representations acquire their “aboutness” are rather varied (Dretske, 1981; Fodor, 1981; Anderson and Rosenberg, 2008), perhaps reflecting the difficulty of grounding the existence of *meaning* (i.e., “aboutness”) in the confines of contemporary naturalism, where “…the harshness of naturalist metaphysics exactly consists in the point that nothing has intrinsic value” (Metzinger, 2017, p. 18).

WST bypasses naturalism's “grounding problem” (Harnad, 1990) because self-sustaining systems emerge *from* the energy transformation *contexts* (i.e., ecologies) in which they sustain themselves (Jordan and Ghin, 2006). As a result, they are naturally and necessarily *about* these contexts. Said another way, self-sustaining systems (i.e., organisms) constitute *embodiments of context*, or *embodied aboutness*. They are aspects of reality (i.e., context) whose activity (i.e., work) gives rise to and sustains a border between the system and the context in which it sustains itself.

According to the notion of *embodied context*, our neuromuscular architecture can be conceptualized as a multi-scale embodiment (i.e., representation) of the constraints that have to be addressed to propel a mass, as a whole, through a gravity field. Given that muscles, bones, and brains constitute *embodiments of context*, WST argues they entail what traditional theories of representation refer to as *representational content* (i.e., “aboutness”). They are embodiments of context that are “about” the contexts they embody.

Given this *embodied-context* approach to “aboutness” versus the traditional *representational-content* approach, WST proposes that *subjectivity, phenomenology*, and *consciousness* constitute forms of embodied aboutness that evolved from lower forms of embodied aboutness such as single- and multi-cell organisms. In the case of a conscious *self*, WST agrees with the SMTS assertion that a PSM, “…generates a pre-attentive self-world border…” (p. 307). According to WST however, the “aboutness” isn't so much an *informational* aspect of a *physical* brain, as it is the contextual emergence of a globally available pattern of neural dynamics whose activity (i.e., “work”) generates and sustains a coherent activation border between itself and the brain as a whole (i.e., activation and inhibition patterns across a large-scale, multi-module network). Consistent with SMTS, WST asserts that the “aboutness” of a conscious *self* comes to be as a particular pattern of self-sustaining neural dynamics emerges within the context of the type of representational (i.e., “aboutness”) context specified by SMTS. Thus, in the end, it seems the biggest difference between SMTS and WST is their ontology, not their science. SMTS begins with a physical-driven naturalism, while WST begins something a bit more Spinozan and conceptualizes all phenomena as embodiments of context, what Spinoza referred to as *finitudes*.

*The glorious YOUs and MEs*. It's glorious being someone. And whether it's an illusion in an inherently meaningless, physical reality, or a perpetually arduous journey through a reality constituted of ubiquitous aboutness, coherence demands that Thomas' SMTS be part of the content entailed in my own self-model of the science of consciousness. Thanks, Thomas, for your hard work. I am a more coherent *someone* because of it.

## Author Contributions

The author confirms being the sole contributor of this work and has approved it for publication.

### Conflict of Interest Statement

The author declares that the research was conducted in the absence of any commercial or financial relationships that could be construed as a potential conflict of interest.
