# Supplementary material for: It's Hard Work Being No One
Source: Front Psychol. 2018 Dec 21;9:2632. doi: 10.3389/fpsyg.2018.02632 (PMC6309154; doi:10.3389/fpsyg.2018.02632)
Supplement: Supplementary file 1 [file Data_Sheet_1.DOCX]

It’s Hard Work *Being No One*

Acknowledgments

When I learned I would be able to upload a recording of “It’s Hard Work *Being No One*“ as part of my contribution, my first task was figuring out how to wipe the grin off of my face. Then, feet firmly back on the ground, I approached Dr. Stephen Parsons, Director of the School of Music at Illinois State University, and asked if he knew of any music students who might be interested in such an opportunity. He put me in contact with Professor Rose Marshack, who invited me to pitch the idea to her music business class. So I visited the class, played the song, explained the lyrics, and asked if any of them would be interested in helping me record it. Shortly thereafter, I received an e-mail from Mr. Isaac Soares. We met for lunch, developed a plan, and within two weeks, made the attached recording.

Isaac recruited two other student musicians to record with us. Thanks, Isaac, for your amazing organizational skills. Isaac played the drum kit, Miles Bohlman played congas and mariba, and Sam Tedeschi played bass guitar. All three students are music majors at Illinois State University. Their bios are listed below.

A fourth student, Derek Zimmerman, who is also a music major at Illinois State University, recorded and mixed the song for us.

Working with these young *someones* was an amazing experience. They immediately grasped the vibe I was after, added their own particular flair, and away we went. I am deeply indebted to these students, as well as the other members of the School of Music who helped make this recording possible.

I’d also like to give a special shout-out to Dr. Jennifer Windt. Jennifer, I am so grateful for your support throughout the entire practice. It is a personal pleasure and a professional honor to work with an editor who is willing to let me express my scholarship in this way. It’s a very lucky *someone* who gets the opportunity to integrate their love of science with their passion for music. I got to be that lucky *someone*. Thank you.

Alles gute!

Scott

"It's Hard Work *Being No One*" was recorded on November 12, 2018 at the Center for Performing Arts, Illinois State University.

The song was written and arranged by J. Scott Jordan, who also sang lead vocals and played rhythm and lead guitar.

Drum Kit:

Isaac Soares

[imsoare@ilstu.edu](mailto:imsoare@ilstu.edu)

 A Junior Music Business major at Illinois State University that is heavily involved in an on-campus record label called Deck Records, and several other organizations such as Music Business Organization and Phi Mu Alpha Sinfonia Fraternity. I am also currently employed by Illinois State University Athletics and serve as the lead Pep Band drummer for all Athletics events. Some goals I have are to network myself and become enveloped in the Music Business industry by working hard and making myself and my work ethic known to those I involve myself with. An end career goal for me would be to work for a production company where I can work behind the scenes planning concerts and organizing events.

Congas and Marimba:

Miles Bohlman was a junior Music Education and Percussion Performance major at Illinois State University when recording this song. At his time at Illinois State University, he participated in the Big Red Marching Machine, the Clef Hanger’s a cappella group, the Percussion Ensemble, and the top Wind Symphony. While at Illinois State, he spent three years working as a drum line and front ensemble instructor with both the Normal Community and Normal Community West high school marching bands. He also was a member of the Cavaliers Indoor Percussion ensemble. He plans to continue his education in Percussion Performance, in hopes of teaching at the collegiate level and performing professionally. He can be reached by email at:[miles.bohlman@gmail.com](mailto:miles.bohlman@gmail.com)

Bass Guitar:

Samuel Tedeschi was a senior Music Education major at Illinois State University at the time of this recording. Throughout his college career, he played the tuba and bass with the top Wind Symphony, Jazz Ensemble, and Jazz Combo. He also spent three years with the Madison Scouts Drum and Bugle Corps. In his years at Illinois State, he had many teaching opportunities in the Bloomington-Normal area and hopes to teach music and marching band back where he grew up in the suburbs of Chicago. He can be reached at his email: [sam.tedeschi14@gmail.com](mailto:sam.tedeschi14@gmail.com)
